# USP15 regulates mitotic fidelity, metastatic potential, and chemotherapeutic response in ovarian cancer cells

**DOI:** 10.1016/j.omton.2026.201329

**Published:** 2026-08-20

**Authors:** Ayokunnumi Ogunsanya, Fatimah Alfaran, Omuya Noel Amadu, Achuth Padmanabhan

**Affiliations:** 1 Department of Biological Sciences, University of Maryland Baltimore County, Baltimore, MD, USA; 2 University of Maryland Greenebaum Comprehensive Cancer Center, Baltimore, MD, USA

**Keywords:** ovarian cancer, USP15, chemoresistance, mitotic fidelity, metastasis, DNA damage, G2-M arrest

## Abstract

Aggressive metastasis and chemoresistance are underlying cause for the poor clinical outcome in ovarian cancer patients. Identifying factors that drive these phenotypes is critical in developing effective therapeutic strategies for metastatic ovarian cancer. In this report, we show that the deubiquitinase USP15 is a critical regulator of ovarian cancer cell proliferation, metastasis, and drug resistance. USP15 levels are elevated in metastatic ovarian tumors and are associated with poor clinical outcome in patients. Depletion and inhibition of USP15 dramatically reduces cell viability and proliferation in a broad spectrum of ovarian cancer cells. Mechanistically, USP15 knockdown disrupts chromosomal segregation, leading to DNA damage, micronuclei formation, and mitotic catastrophe. This causes ovarian cancer cells to undergo cell-cycle arrest at the G2-M checkpoint. Further, USP15 knockdown decreases the metastatic potential of ovarian cancer cells *in vitro*, reduces tumor burden, and extends survival in mouse xenograft models. Consistent with its role in maintaining mitotic fidelity, USP15 depletion sensitizes ovarian cancer cells to mitotic inhibitors and DNA damage inducing chemotherapeutics. Collectively, these findings establish USP15 as a key driver of ovarian cancer metastasis and highlight its potential as an actionable therapeutic target to improve the effectiveness of extant chemotherapeutic agents.

## Graphical abstract

**Figure:**
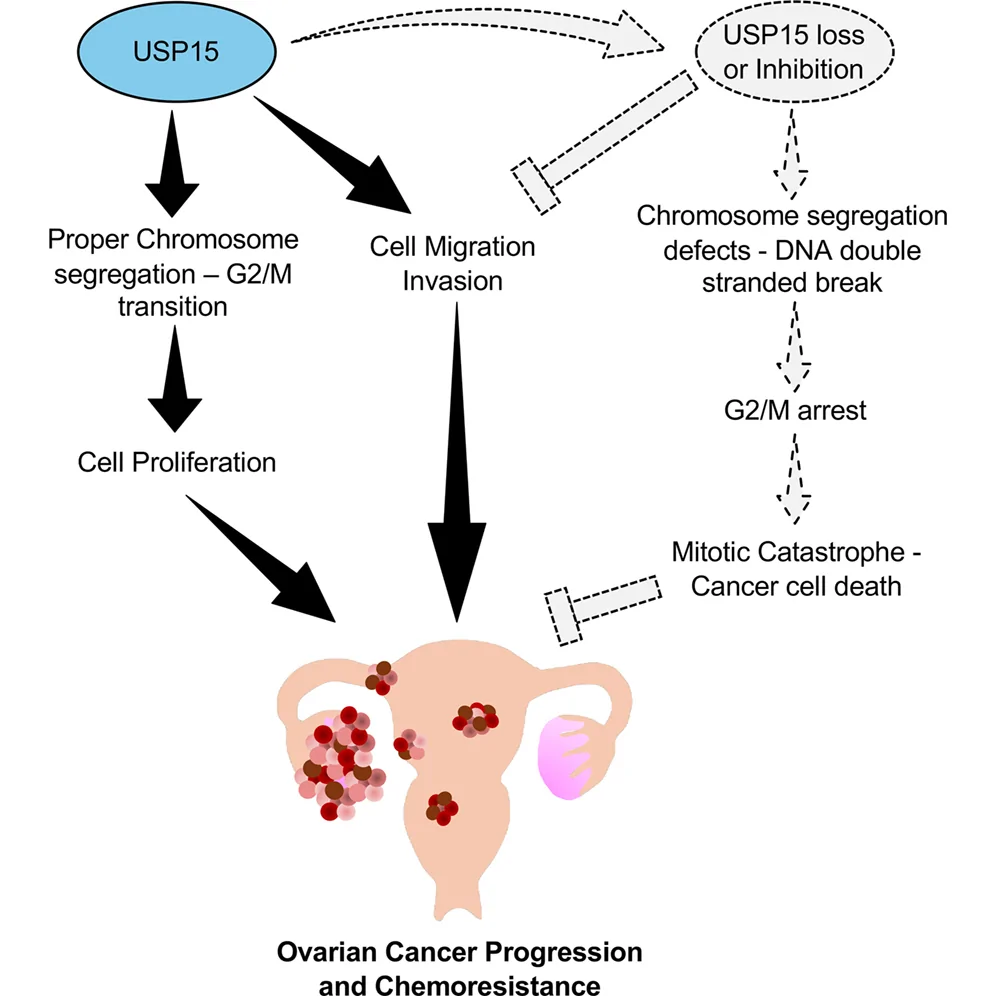

## Teaser

USP15 drives ovarian cancer proliferation, metastasis, and chemoresistance by preserving mitotic fidelity. Its depletion disrupts chromosome segregation, induces DNA damage and G2-M arrest, suppresses metastatic growth, and sensitizes tumors to mitotic inhibitors and DNA-damaging chemotherapy, identifying USP15 as a promising therapeutic target for metastatic ovarian cancer.

## Introduction

Despite recent advances in cancer therapeutics and treatment strategies, ovarian cancer continues to be an extremely lethal disease with a disappointing clinical outcome.^1^ When detected early, extant therapeutics are effective in eliciting a favorable outcome in ovarian cancer patients.^2^ However, due to the lack of reliable early biomarkers and obvious symptoms, over 70% of ovarian cancer cases are diagnosed at an advanced metastatic stage.^3^ First-line treatment for these patients involves cytoreductive surgery followed by chemotherapy using a combination of platinum and taxane-based drugs.^4^^,^^5^ Although most patients respond to this strategy initially, over 85% of patients experience tumor relapse within 6 months.^6^ Unfortunately, relapsed tumors are resistant to extant chemotherapeutics.^5^ Consequently, the 5-year survival rate for metastatic ovarian cancer is less than 30%.^1^ Therefore, to improve clinical outcomes in ovarian cancer patients, it is important to identify factors that drive metastasis and chemoresistance and determine their utility as therapeutic targets.

Recent studies have demonstrated that the levels of the deubiquitinating enzyme Ubiquitin-specific peptidase 15 or Ubiquitin carboxyl-terminal hydrolase 15 (USP15) are elevated in several cancers, such as glioblastoma, multiple myeloma, non-small cell lung cancer (NSCLC), colon cancer, ovarian cancer, and breast cancer.^7^^,^^8^^,^^9^^,^^10^^,^^11^ USP15 knockdown in glioblastoma cells was shown to block metastatic progression.^11^ In NSCLC, USP15 has been shown to impact tumor progression through different mechanisms, such as modulating the TGF-β/SMAD signaling axis, regulating pre-mRNA splicing, and controlling MMP3 and MMP9 levels.^7^^,^^12^^,^^13^ USP15 also facilitates immune escape in NSCLC by regulating PD-L1 stability.^14^ USP15’s ability to deubiquitinate and stabilize the oncogenic HPV16 E6 protein is critical in mediating its oncogenic function in cervical cancer cells.^15^ USP15 protects estrogen receptor ERα from degradation and promotes proliferation in ERα-positive, but not ERα-negative, breast cancer cells.^16^ Thus, USP15 promotes tumor progression and metastasis in different tissues through diverse mechanisms.

USP15 was shown to stabilize the clinically important oncogenic p53-R175H mutant in ovarian cancer cells, implying a potential role in tumors harboring this mutation.^9^ Despite elevated USP15 levels in ovarian cancer cells, its role and impact on ovarian tumor progression remain largely unknown. In this report, we address this critical knowledge gap and establish USP15 as a critical factor that drives both ovarian cancer progression and chemoresistance. Our data show that USP15 impacts both cell cycle progression as well as the metastatic potential of ovarian cancer cells. In addition to impairing metastasis, we show that USP15 depletion sensitizes ovarian cancer cells to extant chemotherapeutics, suggesting its utility as an actionable therapeutic target.

## Results

### USP15 depletion decreases cell viability and proliferation in a broad spectrum of ovarian cancer cells

**Figure 1:**
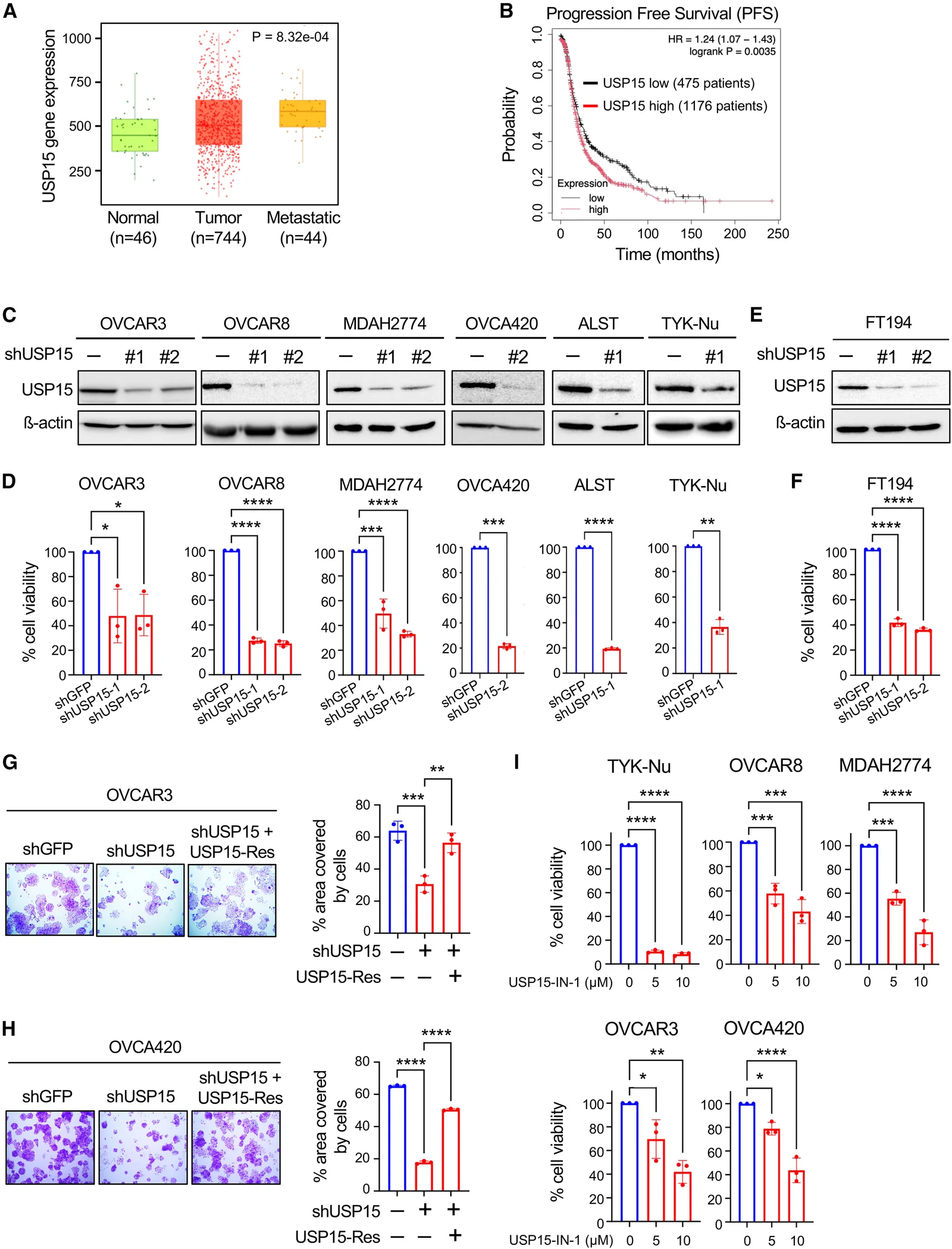
USP15 depletion reduces ovarian cancer cell viability (A) Compared to normal (non-cancerous) ovarian tissue, USP15 mRNA is elevated in ovarian tumors.^17^ Highest USP15 expression is observed in metastatic tumors. (B) Kaplan-Meier analysis showing high USP15 levels in ovarian cancer is associated with a decrease in progression free survival (*n* = 1,651, log rank *p* = 0.0035).^18^ (C) Western blot confirming successful USP15 knockdown in ovarian cancer cell lines. (D) USP15 depletion in ovarian cancer cells causes significant reduction in cell viability and proliferation. Quantified data from *n* = 3 is shown. (E) Western blot showing stable USP15 knockdown in the immortalized fallopian tube secretory epithelial cells, FT194. (F) USP15 depletion in FT194 cells cause significant reduction in cell viability and proliferation. Quantified data from *n* = 3 is shown. (G) Stable expression of shRNA-resistant USP15 rescues the decrease in cell viability observed upon USP15 knockdown in OVCAR3 cells. (H) Stable expression of shRNA-resistant USP15 rescues the decrease in cell viability observed upon USP15 knockdown in OVCA420 cells. (I) USP15 inhibition using the small molecule inhibitor USP15-IN-1 causes significant reduction in cell viability and proliferation in ovarian cancer cells. Quantified data from *n* = 3 is shown.

Analysis of publicly available clinical datasets show that USP15 expression is elevated in ovarian tumors, with highest expression observed in metastatic tumors (Figure 1A).^17^ Interestingly, high USP15 levels in ovarian tumors are associated with poor prognosis (Figure 1B).^18^ These data suggest a potential role for USP15 in driving ovarian cancer progression and metastasis. To determine how USP15 impacts ovarian cancer progression, we used multiple short hairpin RNA (shRNAs) to stably knockdown USP15 in six different ovarian cancer cells: OVCAR3, OVCAR8, OVCA420, MDAH2774, TYK-Nu, and ALST (Figure 1C). USP15 depletion in these cells severely diminished their viability and proliferation (Figures 1D, S1A, and S1B). These results suggest that USP15 depletion impacts cell viability and proliferation in a broad spectrum of genetically distinct ovarian cancer cells. High-grade serous ovarian cancer is known to originate from the secretory epithelial cells of the fallopian tube.^19^ To understand if USP15 could potentially play a role in the early stages of ovarian carcinogenesis, we investigated how USP15 depletion impacts the viability and proliferation in immortalized fallopian tube cells. Similar to ovarian cancer cells, USP15 knockdown in FT194 fallopian tube cells also resulted in a significant reduction in cell viability and proliferation (Figures 1E, 1F, and S1C). Importantly, both short-term and long-term USP15 knockdown did not impact cell viability in the non-cancerous HEK293T cells (Figures S1D–S1F). To ascertain that the phenotype observed upon stably expressing USP15 shRNA in ovarian cancer cells is indeed driven by USP15 depletion, we re-expressed an shRNA resistant form of USP15 in OVCAR3 and OVC420 cells in which USP15 has been previously knocked down using shRNA (Figure S1G). Ectopic expression of shRNA-resistant USP15 rescued the USP15 knockdown-induced decrease in cell viability and proliferation in both OVCAR3 and OVCA420 cells (Figures 1G and 1H). These data confirm that the observed phenotype is indeed due to USP15 depletion.

USP15 is a deubiquitinase and regulates biological processes largely through its catalytic activity. However, USP15 can also impact cellular processes through protein-protein interactions that are independent of its catalytic function. To determine if USP15’s catalytic activity is important to mediate its effect on ovarian cancer cell viability and proliferation, we investigated if USP15 inhibition using a small molecule inhibitor, USP15-IN-1, causes a similar phenotype as USP15 knockdown. USP15-IN-1 treatment also resulted in a dramatic decrease in cell viability and proliferation in TYK-Nu, OVCAR8, MDAH2774, OVCAR3, and OVCA420 cells, suggesting that USP15’s catalytic activity is critical for mediating this effect (Figures 1I and S1H).

**Figure 2:**
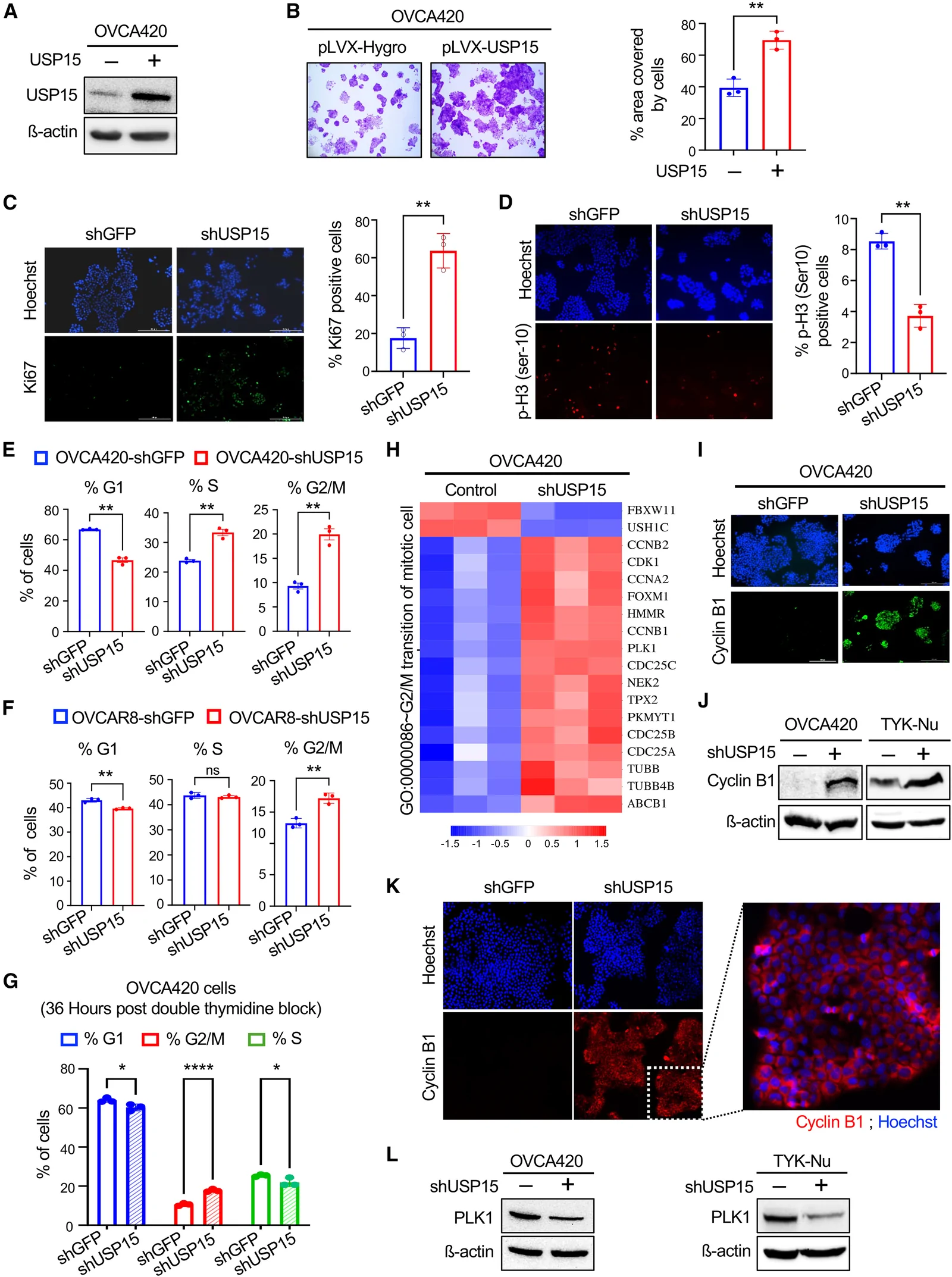
USP15 regulates transition of ovarian cancer cells through the G2-M checkpoint (A) Western blot confirming stable USP15 overexpression in OVCA420 cells. (B) USP15 overexpression causes increase in cell viability and proliferation in OVCA420 cells. Quantified data from *n* = 3 is shown. (C) Immunofluorescence data reveal elevated Ki67 staining in OVCA420 cells upon USP15 knockdown. Quantified data from *n* = 3 is also shown. (D) Immunofluorescence data show reduction in phospho-histone H3 (Ser10) staining in OVCA420 cells upon USP15 knockdown. Quantified data from *n* = 3 is also shown. (E) Cell-cycle analysis using sub-confluent OVCA420 cells show increase in G2-M population in USP15 knockdown cells. Quantified data from *n* = 3 is also shown. (F) Cell-cycle analysis using sub-confluent OVCAR8 cells show increase in G2-M population in USP15 knockdown cells. Quantified data from *n* = 3 is also shown. (G) Cell-cycle analysis using synchronized OVCA420 cells show increase in G2-M population in USP15 knockdown cells. Quantified data from *n* = 3 is also shown. (H) Gene Ontology analysis in OVCA420 cells reveal that genes that mediate G2-M transition is altered upon USP15 knockdown. (I) Immunofluorescence reveal elevated Cyclin B1 levels in sub-confluent OVCA420-USP15 knockdown cells. (J) Western blot analysis shows higher Cyclin B1 levels in USP15-depleted OVCA420 and TYK-Nu cells. (K) Immunofluorescence data showing cytoplasmic accumulation of Cyclin B1 in OVCA420-USP15 knockdown cells 36 h post double thymidine block (synchronization). (L) Western blot analysis showing PLK1 levels are reduced upon USP15 knockdown in OVCA420 (left) and TYK-Nu (right) cells.

Having demonstrated that USP15 depletion compromises ovarian cancer cell viability and proliferation, we next investigated whether USP15 overexpression exerts the opposite effect. To address this, we generated OVCA420 cells stably expressing USP15 and assessed their proliferative capacity (Figure 2A). Consistent with the inhibitory effects observed following USP15 depletion or pharmacologic inhibition in ovarian cancer cells, ectopic USP15 overexpression significantly enhanced proliferation in OVCA420 cells (Figure 2B). Therefore, these data taken together establish USP15 as a positive regulator of ovarian cancer cell growth and viability.

### USP15 regulates the transit of ovarian cancer cells through the G2-M checkpoint

To understand how USP15 depletion is impacting ovarian cancer cell viability, we determined the effect of USP15 knockdown on cell proliferation markers, such as Ki67 and phospho-histone H3 (Ser10). Surprisingly, despite USP15 depletion causing a significant reduction in the number of ovarian cancer cells in the cell proliferation assay, we saw a profound increase in Ki67 staining in USP15 knockdown cells (Figure 2C). However, fewer USP15 knockdown cells were positive for phospho-histone H3 compared with control cells (Figure 2D). An increase in Ki67 combined with a reduction in phospho-histone H3 suggested that USP15 depletion causes these cells to undergo cell-cycle arrest at the G2-M checkpoint. To determine if this is indeed the case, we performed cell-cycle analysis using propidium iodide (PI) staining on sub-confluent control and USP15 knockdown OVCA420 and OVCAR8 cells. USP15 depletion in these cells resulted in an increase G2-M population (Figures 2E and 2F). Similar results were also obtained using synchronized OVCA420 cells (Figure 2G). RNA sequencing (RNA-seq) analysis revealed that several genes that are critical for G2-M transition in mitotic cells are significantly altered upon USP15 depletion in both OVCA420 and TYK-Nu cells, confirming that alterations at the molecular level are consistent with the observed phenotype (Figures 2H and S2A). An increase in Cyclin B1 levels, a hallmark of G2-M arrest, was observed at the mRNA level in USP15 KD cells in the RNA-seq data (Figure 2H).^20^ Consistently, elevated Cyclin B1 protein level is observed in ovarian cancer cells upon USP15 depletion (Figures 2I and 2J). During normal cell cycle, Cyclin B1 translocates from the cytoplasm to the nucleus to initiate mitosis.^21^ However, when cell cycle is arrested at the G2-M checkpoint in response to DNA damage or checkpoint activation, Cyclin B1 is sequestered in the cytoplasm, preventing its interaction with nuclear substrates.^21^ Immunofluorescence analysis using synchronized OVCA420 cells (36 h after double thymidine block), show that in addition to Cyclin B1 levels being elevated, Cyclin B1 is also retained in the cytoplasm in USP15 depleted OVCA420 cells (Figure 2K). Interestingly, the levels of the mitotic protein kinase Plk1 were significantly reduced in USP15-depleted OVCA420 and TYK-Nu cells (Figure 2L). Plk1 is crucial for mitotic entry following DNA damage.^22^^,^^23^ A decrease in Plk1 protein levels during G2-M arrest is an indicator of DNA damage response checkpoint activation, a critical step that prevents cells with damaged DNA from entering mitosis.^22^^,^^23^ Therefore, these results demonstrate that USP15 knockdown induces G2-M arrest in ovarian cancer cells, potentially through the induction of DNA damage.

### USP15 depletion induces DNA damage, micronuclei formation, and mitotic catastrophe

**Figure 3:**
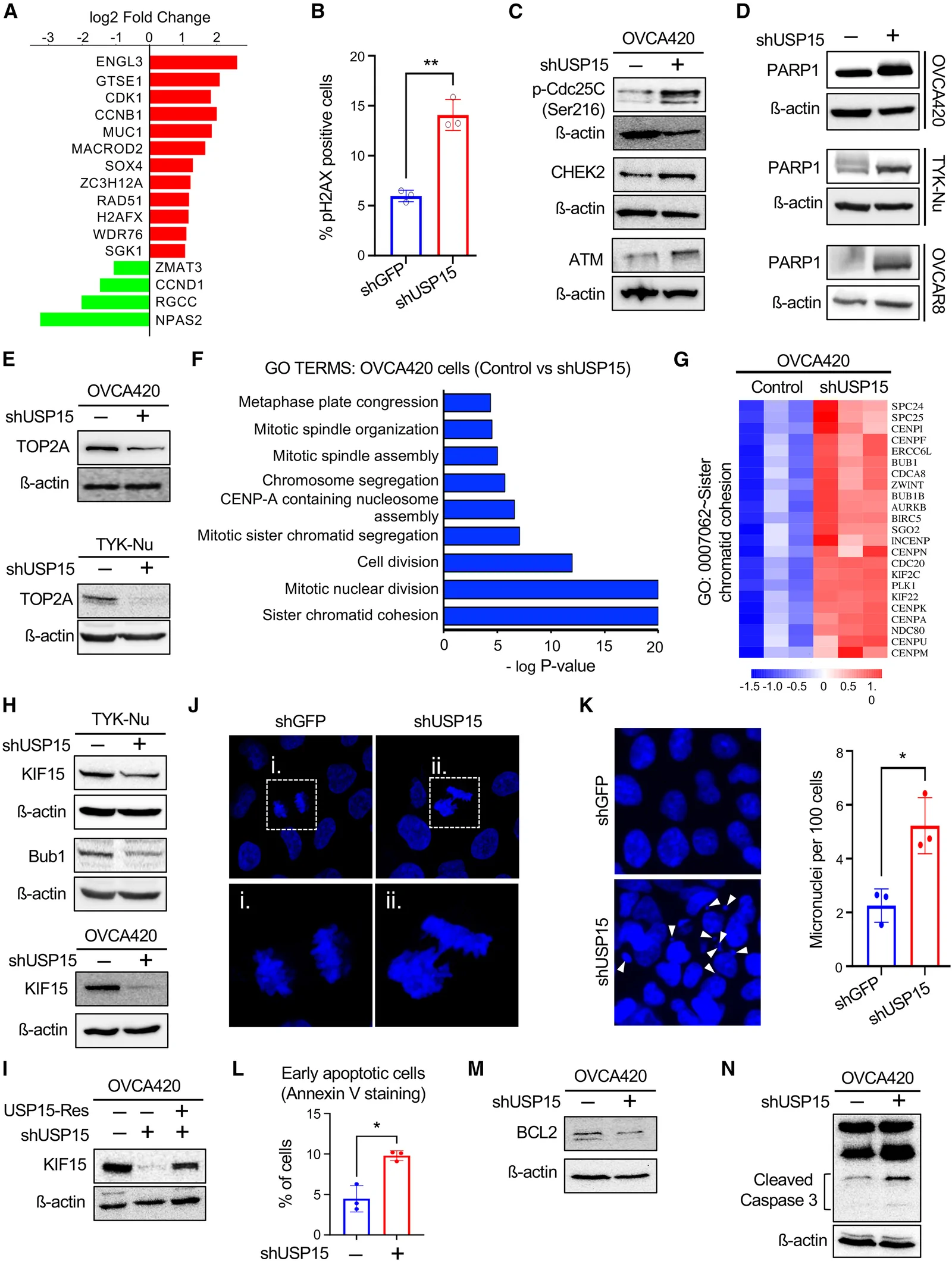
USP15 depletion induces chromosome segregation defects and DNA damage (A) RNA-seq analysis revealed that several genes involved in DNA damage response is altered upon USP15 knockdown in OVCA420 cells. (B) USP15 depletion in OVCA420 cells caused an increase in γH2AX positive cells. Quantification for *n* = 3 is shown. (C) Western blot showing USP15 knockdown in OVCA420 causes an increase in markers of DNA damage such as phospho-Cdc25C, CHEK2, and ATM. (D) Western blot showing PARP1 accumulates in OVCA420, TYK-Nu, and OVCAR8 cells upon USP15 knockdown. (E) Western blot showing USP15 depletion results in reduction in TOP2A levels in OVCA420 and TYK-Nu cells. (F) Gene Ontology analysis using USP15-depleted OVCA420 cells reveal alterations in cellular processes involved in spindle assembly and chromosome segregation. (G) Heatmap showing genes that are differentially altered in control and USP15 knockdown OVCA420 cells that are involved in sister chromatid cohesion. (H) Western blot showing USP15 knockdown causes decrease in KIF15 and Bub1. (I) Stable expression of shRNA-resistant USP15 rescues the decrease in KIF15 observed upon USP15 knockdown in OVCA420 cells. (J) USP15 knockdown in OVCA420 cells results in the formation of anaphase bridges. (K) USP15 knockdown increases the number of micronuclei in OVCA420 cells. Quantified data from *n* = 3 are shown on the right. (L) Annexin V staining shows an increase in early apoptotic cells upon transient USP15 knockdown (6 days) in TYK-Nu cells. (M) Transient USP15 knockdown in OVCA420 causes a decrease in BCL2 levels. (N) Transient USP15 knockdown in OVCA420 causes an increase in the level of cleaved caspase-3.

G2-M arrest is often associated with mitotic defects and DNA damage.^24^ The induction of G2-M arrest with a simultaneous reduction in Plk1 levels suggested that USP15 depletion is potentially causing DNA damage in these cells. Analysis of RNA-seq data from control and USP15 knockdown OVCA420 and TYK-Nu cells also revealed alterations in several genes associated with cellular response to DNA damage (Figures 3A and S3A). Consistent with increased DNA double-stranded breaks, γH2AX was significantly elevated in USP15-depleted OVCA420 cells (Figures 3B and S3B). USP15 depletion in ovarian cancer cells resulted in pronounced activation of several components of the DNA damage response pathway and G2-M arrest, such as phospho-Cdc25C (Ser216), ATM, and CHEK2, were elevated upon USP15 knockdown (Figure 3C). Ectopic expression of shRNA-resistant USP15 in OVCA420 cells stably expressing USP15 shRNA reversed the increase in Cdc25C phosphorylation, confirming specificity of USP15 shRNA (Figure S3C). PARP1 accumulation is a critical early step in DNA damage response.^25^ Again, consistent with the observed phenotype, significant upregulation of PARP1 was observed in USP15 depleted ovarian cancer cells (Figure 3D).

During the G2-M phase of the cell cycle, high TOP2A activity is important to ensure proper chromosome segregation.^26^^,^^27^ USP15 knockdown resulted in reduced TOP2A levels, suggesting the potential for chromosome segregation defects in these cells (Figure 3E). This was further supported at the molecular level by RNA-seq data showing alterations in multiple cellular processes associated with mitotic spindle assembly, sister chromatid cohesion, and chromosome segregation (Figures 3F, 3G, and S3D). KIF15 (kinesin-12) is a plus-end-directed motor protein essential for establishing and maintaining bipolar spindle integrity during mitosis, ensuring proper chromosome alignment, and segregation.^28^ USP15 knockdown resulted in a dramatic decrease in KIF-15 levels in ovarian cancer cells (Figure 3H). Similarly, USP15 depletion also reduced the levels of the serine-threonine protein kinase Bub1, which is critical for ensuring faithful chromosome segregation during mitosis (Figure 3H). Bub 1 plays an important role in delaying anaphase until chromosomes are aligned and in ensuring accurate attachment of kinetochores to spindle microtubules.^29^^,^^30^ Ectopic expression of shRNA-resistant USP15 in OVCA420 cells stably expressing USP15 shRNA rescued the depletion of KIF15 confirming specificity of USP15 shRNA (Figure 3I). Consistent with these changes at the molecular level in components regulating the proper segregation of chromosomes during mitosis, USP15 knockdown cells exhibit defects in chromosome segregation as evidenced by the formation of anaphase bridges (Figure 3J). These chromatin-bridges formed during anaphase eventually rupture, resulting in DNA breakage.^31^^,^^32^ Lagging chromosome from these broken anaphase bridges that fail to be included in the main nucleus are enveloped in their own nuclear membrane, creating micronuclei.^33^ A significantly higher number of micronuclei were observed in ovarian cancer cells upon USP15 knockdown (Figure 3K). As these molecular changes usually cause cells to undergo programmed cell death, we next investigated if USP15 depletion induces apoptosis in ovarian cancer cells by examining the effect of transient USP15 knockdown in ovarian cancer cells on multiple apoptotic markers. Annexin V staining followed by flow cytometry demonstrated an increase in the proportion of early apoptotic TYK-Nu cells following USP15 knockdown (Figure 3L). Consistent with this observation, USP15 depletion resulted in reduced levels of the anti-apoptotic protein BCL2 (Figure 3M). Apoptosis was further confirmed by western blot analysis using a caspase-3 antibody (Figure 3N), which showed increased levels of cleaved caspase-3 in USP15-depleted cells compared with control cells expressing GFP-targeting shRNA. Taken together, USP15 depletion impacts multiple proteins involved in the proper segregation of chromosomes during anaphase, leading to DNA damage and induction of mitotic catastrophe in ovarian cancer cells.

### USP15 depletion decreases the metastatic potential of ovarian cancer cells *in vitro*

**Figure 4:**
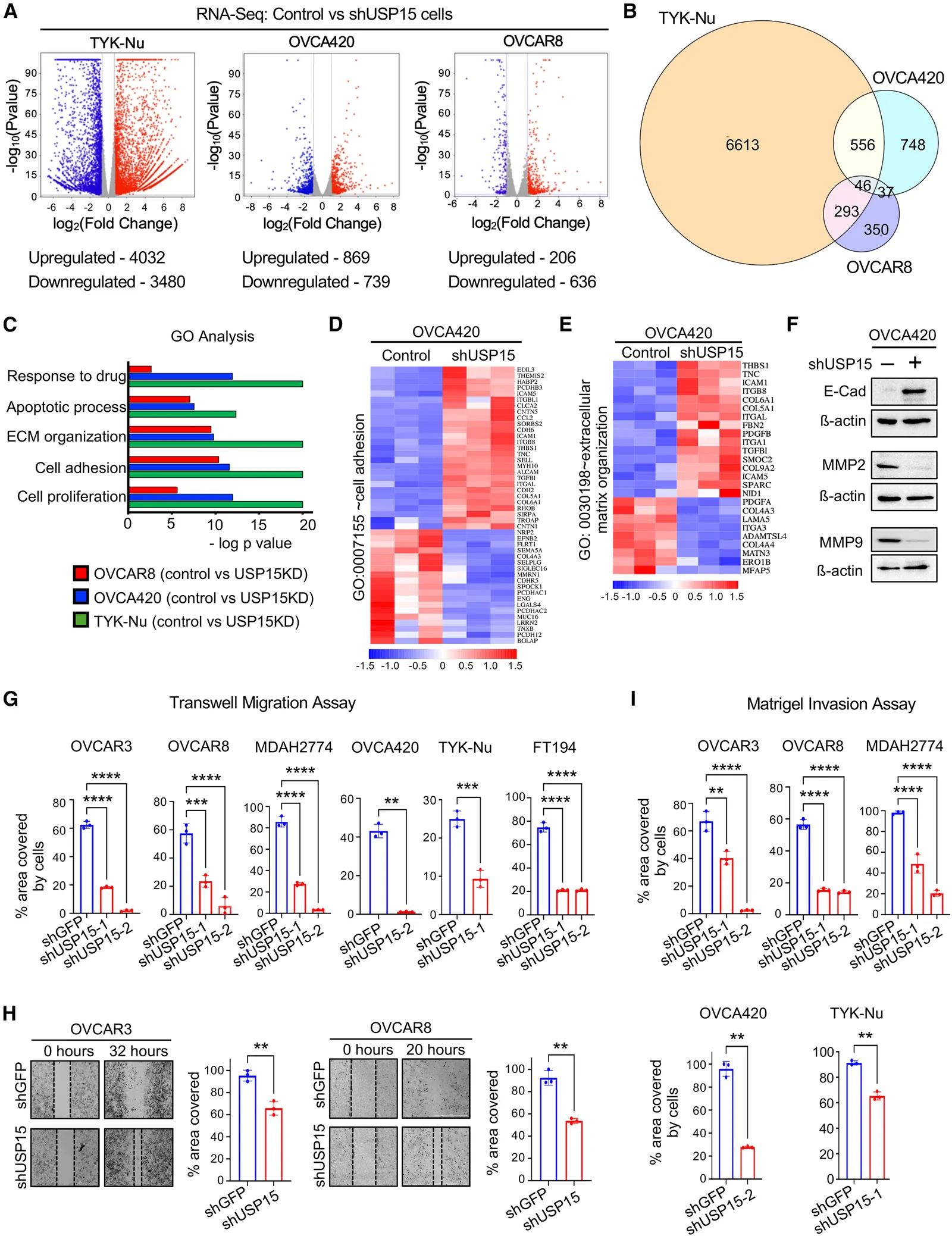
USP15 impacts the metastatic potential of ovarian cancer cells (A) Volcano plot showing differentially regulated genes in USP15 knockdown TYK-Nu, OVCA420, and OVCAR8 cells (*n* = 3; *p* value < 0.05; log2 fold change >1). (B) Venn diagram showing number of differentially regulated genes that were unique and shared between control and USP15 knockdown TYK-Nu, OVCA420, and OVCAR8 cells. (C) Gene Ontology terms that are significantly enriched in the differentially expressed gene sets between control and USP15 knockdown in TYK-Nu, OVCA420, and OVCAR8 cells. (D) Heatmap showing differentially regulated genes in USP15-depleted OVCA420 cells related to regulation of cell adhesion. (E) Heatmap showing differentially regulated genes in USP15-depleted OVCA420 related to ECM organization. (F) Western blot show EMT markers are altered upon USP15 knockdown in OVCA420. (G) Transwell migration assay show that USP15 depletion reduces ovarian cancer cell migration (*n* = 3). (H) USP15 depletion reduces migration of ovarian cancer cells in wound healing assay (*n* = 3). (I) USP15 knockdown reduces the ability of ovarian cancer cells to invade through Matrigel (*n* = 3).

We next investigated if USP15 can impact ovarian cancer progression through mechanisms beyond regulating the transition of cancer cells through the G2-M checkpoint. To address this question, we performed RNA-seq using control and USP15 knockdown cells from three different ovarian cancer cell lines—TYK-Nu, OVCA420, and OVCAR8. USP15 depletion resulted in differential regulation of 7,512 genes in TYK-Nu, 1,608 genes in OVCA420, and 842 genes in OVCAR8 cells (Figures 4A; Tables S1, S2 and S3). A significant percentage of genes that were differentially expressed upon USP15 knockdown in OVCA420 (37%) and OVCAR8 (40%) cells were also altered in TYK-Nu cells (Figures 4B; Tables S4, S5 and S6). In addition to regulating cell proliferation, Gene Ontology (GO) analysis revealed several key processes such as cell adhesion, extracellular matrix (ECM) organization, and response to drugs is altered upon USP15 depletion in all three ovarian cancer cell lines (Figures 4C–4E). As these processes are associated with metastatic potential of cancer cells, we determined if USP15 depletion impacted epithelial versus mesenchymal state of ovarian cancer cells. USP15 knockdown resulted in an increase in the epithelial marker E-cadherin suggesting a potential mesenchymal-to-epithelial shift in cellular state upon USP15 depletion (Figure 4F). USP15 depletion also decreased the levels of ECM remodeling enzymes MMP2 and MMP9, suggesting a reduction in their metastatic potential (Figure 4F). Consistent with these changes, USP15 knockdown resulted in a significant reduction in cell migration in a broad spectrum of ovarian cancer cells such as OVCAR3, OVCAR8, MDAH2774, OVCA420, and TYK-Nu (Figures 4G and S4A). As high-grade serous ovarian cancer cells are known to originate from the fallopian tube secretory epithelial cells, we investigated how USP15 impacts metastatic properties in cells that are involved in the early stages of disease progression. As with ovarian cancer cells, USP15 knockdown in FT194 cells also resulted in a dramatic reduction in the ability of these cells to migrate in the transwell assay (Figures 4G and S4A). We used the wound healing assay to further confirm that USP15 depletion reduces the ability of ovarian cancer cells to migrate *in vitro* (Figure 4H). Cancer cells need to remodel and invade through the ECM to successfully metastasize through tissues. To understand how USP15 impacts this process, we determined the effect of USP15 knockdown on the ability of ovarian cancer cells to invade through the ECM-like Matrigel *in vitro*. USP15 knockdown significantly reduced the ability of a diverse panel of ovarian cancer cells to invade through the Matrigel (Figures 4I and S4B). These results are again consistent with the fact that USP15 depletion is associated with a more epithelial phenotype and reduction in expression of ECM remodeling enzymes such as MMP2 and MMP9 (Figure 4F).

### USP15 depletion decreases ovarian tumor burden and improves survival *in vivo*

**Figure 5:**
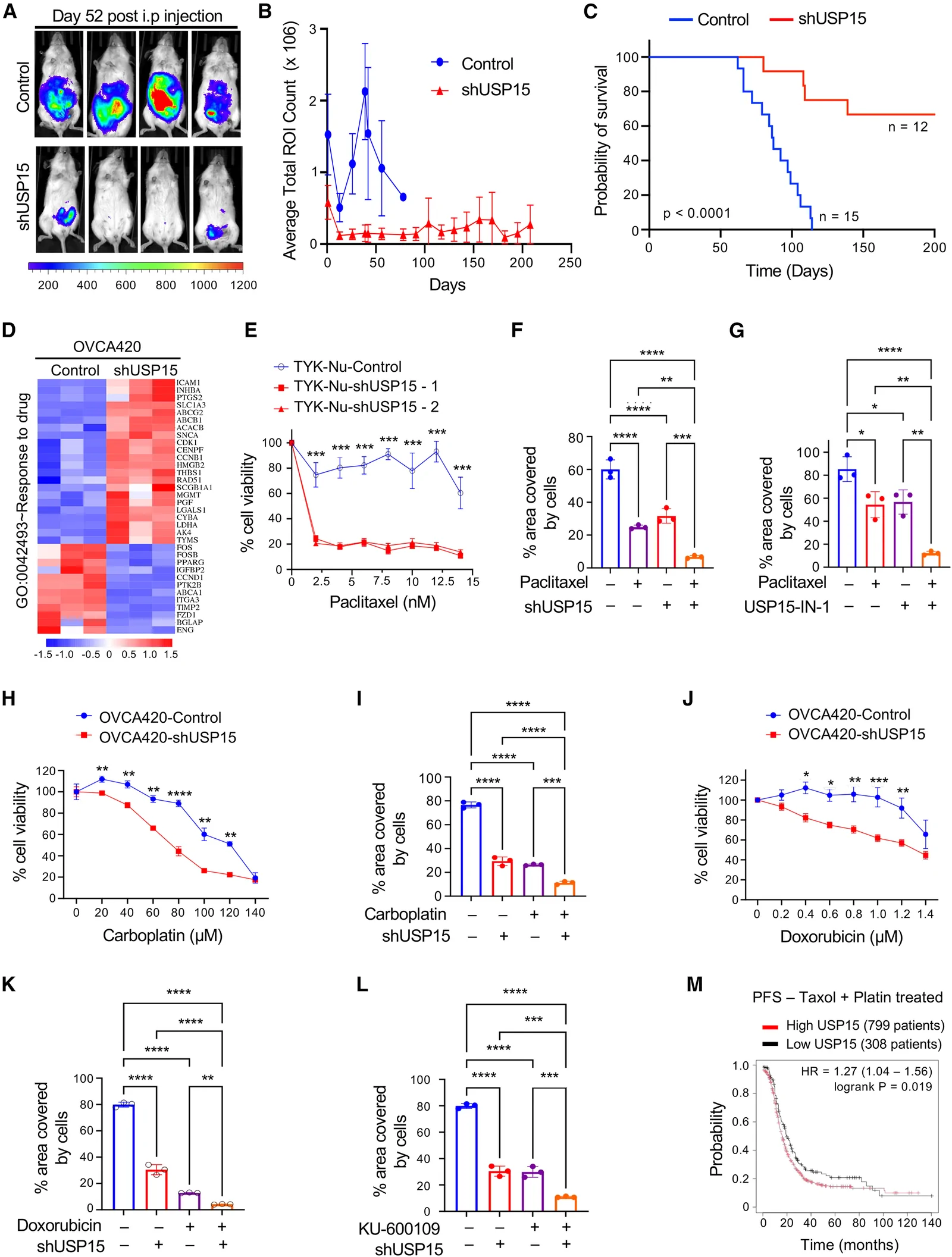
USP15 depletion sensitizes ovarian cancer cells to chemotherapeutics (A) Luciferase signal on day 52 post i.p. injection shows that USP15 knockdown in TYK-Nu cells impairs metastatic tumor burden in NSG mice. (B) Quantification of *in vivo* imaging in NSG mice show that USP15 knockdown in TYK-Nu cells impairs metastatic tumor progression in NSG nude mice upon i.p. injection (*n* = 4). (C) Kaplan–Meier curve showing that USP15 depletion in TYK-Nu cells (*n* = 12) causes an increase in survival compared to control (*n* = 15) in an i.p. injection model of metastatic ovarian cancer in NSG mice (*p* = 0.0054). (D) Gene Ontology analysis from bulk RNA-seq of OVCA420 cells revealed deregulation of genes implicated in drug response in USP15 knockdown cells. Heatmap showing the differentially regulated genes that are related to drug response. (E) WST-1 cell viability assay (48 h) shows that USP15 knockdown sensitizes TYK-Nu cells to paclitaxel (*n* = 3). The IC_50_ values are as follows: TYK-Nu control >14 nM; TYK-Nu shUSP15-1 = 0.3772 nM; TYK-Nu shUSP15-2 = 0.8255 nM. (F) Quantified data from crystal violet staining show that USP15 knockdown TYK-Nu cells exhibit increased sensitivity to paclitaxel even upon treatment for a longer duration (0.5 nM; 5 days; *n* = 3). (G) Quantified data from crystal violet staining show that USP15 inhibitor (USP15-IN-1, 2.5 μM) increases the sensitivity of TYK-Nu cells to paclitaxel (0.5 nM; 6 days; *n* = 3). (H) WST-1 cell viability assay (48 h) shows that USP15 knockdown sensitizes OVCA420 cells to carboplatin (*n* = 3). The IC_50_ values are as follows: OVCA420 control = 113.4 μM; OVCA420 shUSP15 = 74.79 μM. (I) Quantified data from crystal violet staining show that USP15 knockdown TYK-Nu cells sensitizes these cells to carboplatin (10 μM; 5 days; *n* = 3). (J) WST-1 cell viability assay (72 h) shows that USP15 knockdown sensitizes OVCA420 cells to doxorubicin (*n* = 3). The IC_50_ values are as follows: OVCA420 control >1.4 μM; OVCA420 shUSP15 = 1.359 μM. (K) Quantified data from crystal violet staining show that USP15 knockdown TYK-Nu cells sensitizes these cells to doxorubicin (100 nM; 5 days; *n* = 3). (L) Quantified data from crystal violet staining show that USP15 knockdown TYK-Nu cells sensitizes these cells to KU-600109 (5 μM; 5 days; *n* = 3). (M) Data retrieved from KMplotter showing that ovarian cancer patients with high USP15 levels in their tumor respond poorly to chemotherapy containing taxol and platin-based compounds.

To determine how USP15 depletion impacts the ability of ovarian cancer cells to form metastatic tumors *in vivo*, we used an intraperitoneal (i.p.) mouse xenograft model. The i.p injection of cancer cells closely mimics the physiological route and microenvironment through which ovarian cancer cells metastasize and is widely used as a clinically relevant model to study ovarian cancer metastasis.^34^^,^^35^ Control and USP15 knockdown TYK-Nu cells stably expressing luciferase were injected i.p. into 6- to 8-week-old female NSG mice. Stable luciferase expression in these cells allowed non-invasive longitudinal monitoring of tumor progression. USP15 knockdown in TYK-Nu cells resulted in a significant reduction in their ability to form metastatic tumors in mice (Figures 5A, 5B, and S5A). As expected with reduction in tumor burden, mice bearing USP15-depleted tumors exhibited a profound increase in survival compared to mice injected with control cells (Figure 5C). The median survival of mice injected with parental TYK-Nu cells was 87 days (Figure 5C). In comparison, the median survival of mice injected with USP15 knockdown TYK-Nu cells was 300 days (Figure 5C). Thus, our *in vitro* and *in vivo* data taken together establish USP15 as an important factor that regulates ovarian cancer progression, metastasis, and survival outcome.

### USP15 depletion sensitizes ovarian cancer cells to chemotherapeutics

RNA-seq data from control and USP15 knockdown cells in multiple ovarian cancer cell lines suggested alterations in drug response pathways (Figures 4C, 5D, and S5B). Further, because USP15 knockdown impacts mitotic fidelity and DNA damage repair pathways, we hypothesized that targeting USP15 will sensitize ovarian cancer cells to chemotherapeutic drugs that impact these processes. As expected, USP15 depletion in TYK-Nu cells increased their sensitivity to the microtubule-targeting drug paclitaxel upon both short-term treatment (48 h) as well as low dose treatment for longer time period (0.5 nM; 5 days) (Figures 5E, 5F, and S5C). Similar effect was observed using the USP15 inhibitor USP15-IN-1 in combination with paclitaxel (Figures 5G and S5D). USP15 depletion also sensitized both OVCA420 and TYK-Nu cells to drugs that induce DNA damage such as carboplatin (Figures 5H, 5I, and S5E) and doxorubicin (Figures 5J, 5K, and S5F). As ATM is a key protein kinase involved in repairing damaged DNA, we investigated if USP15 knockdown sensitizes ovarian cancer cells to the ATM inhibitor KU60019. As expected, USP15 knockdown increased the sensitivity of TYK-Nu cells to KU60019 (Figures 5L and S5G). Therefore, USP15 depletion synergizes with chemotherapeutic agents that impairs microtubule dynamics or interferes with DNA damage response mechanisms to induce ovarian cancer cell killing. Consistent with these effects, analysis of clinical data reveals that ovarian cancer patients with elevated USP15 expression in their tumors respond poorly to taxol and platinum-containing drugs (Figure 5M). These drugs, such as paclitaxel and carboplatin, respectively are frontline chemotherapeutics used to treat metastatic ovarian cancer. Collectively, our data suggests that USP15 is a novel actionable therapeutic target that can improve the effectiveness of extant chemotherapeutic agents used to treat metastatic ovarian cancer.

## Discussion

**Figure 6:**
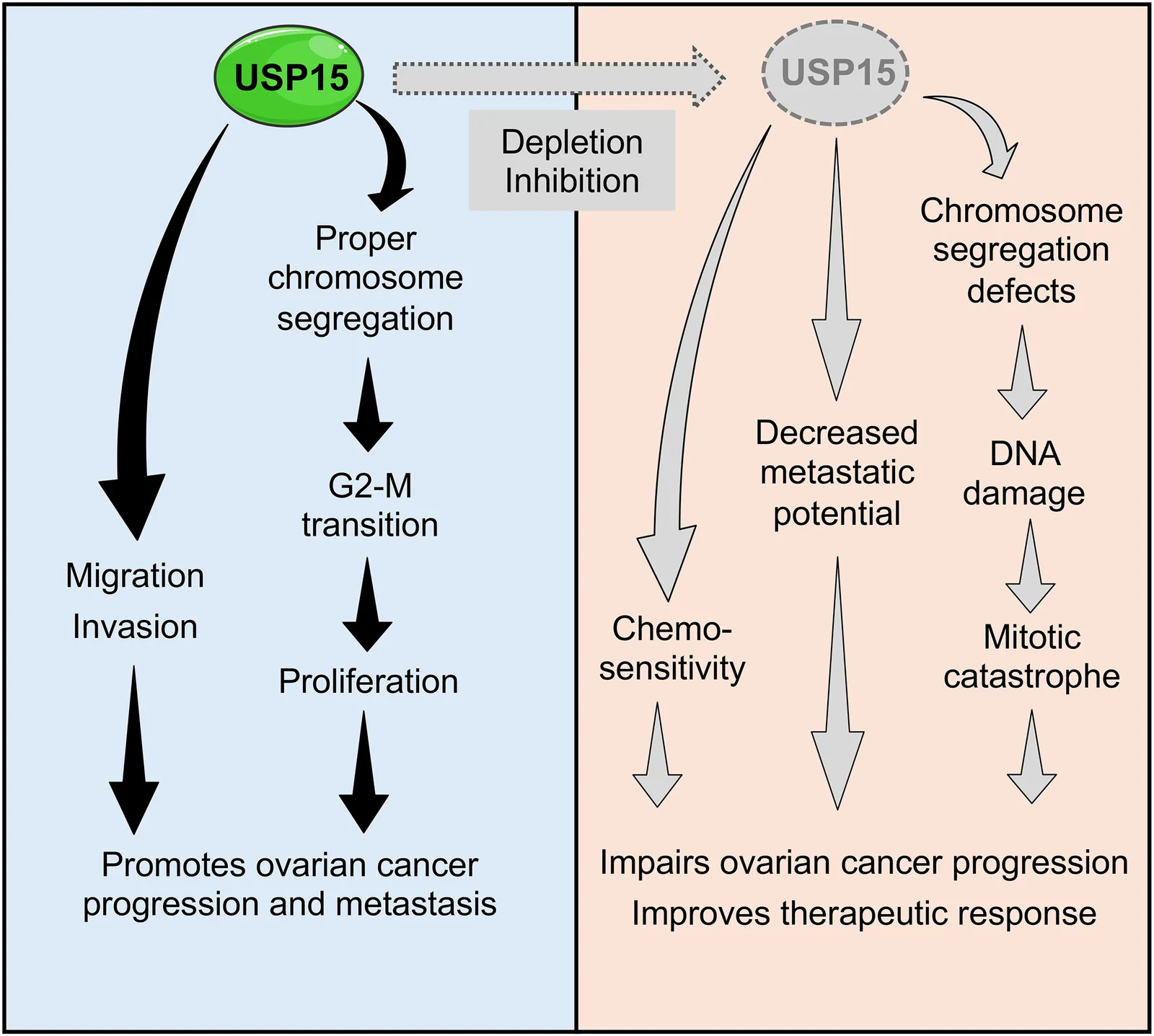
Summary of key findings of this study USP15 regulates mitotic fidelity, transition of cells through the G2-M phase, metastatic potential, and chemoresistance in ovarian cancer cells.

Understanding factors that drive ovarian cancer metastasis and chemoresistance is critical for developing actionable therapeutic targets that can improve clinical outcome. In this report, we show that the deubiquitinase USP15 regulates cell proliferation, metastatic potential, and therapeutic response in a panel of genetically diverse ovarian cancer cells (Figure 6). USP15 depletion in ovarian cancer cells causes defects in chromosome segregation during anaphase, leading to double-stranded breaks in DNA and cell-cycle arrest at the G2-M checkpoint (Figure 6). Further, USP15 knockdown severely impaired the metastatic potential of ovarian cancer cells. In line with its role in maintaining mitotic fidelity and regulating DNA damage response, depleting USP15 level or activity enhances the sensitivity of ovarian cancer cells to extant chemotherapeutic agents that disrupt these processes (Figure 6). These results suggest that strategies that target USP15 have the potential to delay tumor progression and overcome chemoresistance in a broad spectrum of ovarian cancer patients. As these processes are important to all cancer cells, the benefits of targeting USP15 are likely to extend well beyond ovarian cancer.

In addition to unraveling the mechanisms through which USP15 impacts ovarian cancer progression, our work establishes USP15 as an attractive therapeutic target in ovarian cancer cells. Resistance to carboplatin and paclitaxel, the frontline therapeutics used to treat metastatic ovarian cancer, is a major therapeutic challenge contributing to poor clinical outcome. Our data using ovarian cancer cells show that targeting USP15 will improve the response to these drugs. Other studies have also highlighted the untapped potential of USP15 as an anti-cancer therapeutic target. For instance, USP15 was shown to regulate response to PARP inhibitors by regulating homologous recombination.^36^ In this report, they show that in breast cancer and osteosarcoma cells, USP15 is recruited to sites of double-stand DNA breaks, where they promoted BARD1/BRCA1 retention at these sites.^36^ USP15 depletion resulted in increased sensitivity to PARP inhibitors even in BRCA wild-type cells such as MCF7.^36^ These findings are consistent with our discovery that USP15 regulates DNA damage response in ovarian cancer cells. In addition to promoting tumor progression through cancer cell intrinsic functions, USP15 also impacts the interaction between cancer cells with the immune cells in the tumor microenvironment. USP15 stabilizes CTLA4, a key immune checkpoint receptor, and enhances its expression on the cell surface.^37^ Further, USP15 expression in immune cells was also shown to regulate the ability of immune cells to mount anti-tumor response. Specifically, USP15 negatively regulates T cell receptor signaling in CD4^+^ T cells and USP15 depletion promotes naive CD4^+^ T cell activation and differentiation into IFN-γ-producing Th1-cells.^38^ In contrast to its effect on CD4^+^ T cells, USP15 does not directly regulate CD8^+^ T cells.^38^ However, USP15 deficiency enhanced CD8^+^ T cell responses *in vivo*, likely due to the stronger helper function of Th1 cells.^38^ Overall, USP15 depletion promoted T cell activation *in vitro* and enhanced anti-tumor T cell response in mice, suggesting a key role for USP15 in tumor immune microenvironment (TIME) and immunotherapeutic response.^38^ Therefore, targeting USP15 could potentially exert anti-tumor effects through both cancer cell intrinsic as well as extrinsic mechanisms.

A clinical challenge with miotic inhibitors such as paclitaxel is mitotic slippage-induced resistance.^39^^,^^40^ In cells undergoing mitotic slippage, the spindle assembly checkpoint fails, and the cells that are arrested in mitosis exit mitosis without dividing.^40^ Consequently, they escape cell death and form polyploid or multi-nucleated interphase cells that are resistant to these drugs.^40^ Overcoming mitotic slippage during chemotherapy is important to prevent emergence of drug resistance and can be achieved by combining strategies that target multiple proteins that are important in mitotic fidelity. Based on our data, targeting USP15 in combination with mitotic inhibitors such as paclitaxel and KU-600109 will be an exciting approach to address this issue. Combining USP15 inhibition with mitotic inhibitors should also help with reducing the dosage of these drugs, thereby limiting off-target effects.

USP15 plays an important role in several biological processes such as cell cycle progression, apoptosis, autophagy, DNA damage repair, maintenance of genomic integrity, and immune response.^41^ Therefore, one concern is the impact USP15 depletion or inhibition will have on non-tumor cells. However, despite its role in important cellular processes, USP15 knockout mice are viable, suggesting that USP15 can be targeted therapeutically.^36^ As with most chemotherapeutics, realizing USP15’s therapeutic potential will depend on finding an appropriate therapeutic window. Another challenge in clinical translation of USP15 as a therapeutic target is the inherent difficulty in targeting deubiquitinases selectively due to their high structural similarity.^42^^,^^43^ This issue can be overcome by developing USP15-specific PROTACs or by targeting upstream regulators of USP15.

Although USP15 levels are altered in several tumors, including ovarian cancer, upstream factors that regulate USP15 abundance in cells remain poorly understood. While changes in USP15 mRNA levels can contribute to protein abundance, post-transcriptional mechanisms can also contribute to changes in USP15 levels. Further, in the case of an enzyme such as USP15, changes in activity can be mediated by mechanisms that are independent of their abundance. Future investigation into these questions is critical for understanding how USP15 regulates tumor progression and for developing novel strategies to target USP15. Our data shows that USP15 impacts oncogenic phenotypes in fallopian tube epithelial cells. As high-grade serous ovarian cancer is known to originate from the secretory epithelial cells in the fallopian tube, it will also be important to understand if USP15 levels or activity are altered during early stages of ovarian carcinogenesis, such as in serous intraepithelial carcinomas or STIC lesions. These studies will inform us if USP15 can serve as a therapeutic target for early intervention in ovarian cancer. Taken together, our findings establish USP15 as an important factor driving ovarian cancer progression and a therapeutic target with immense potential.

## Materials and methods

### Plasmids and cloning

Lentiviral envelope and packaging vectors, pMD2.G (Plasmid #12259) and psPAX2 (Plasmid #12260), were obtained from Addgene. Validated USP15 shRNA in the lentiviral vector pLKO.1-puro were purchased from Sigma-Aldrich (Mission lentiviral shRNA). The sequence of USP15 shRNAs used in this study is the following: shUSP15-1, CCTTGGAAGTTTACTTAGTTA; shUSP15-2, GCTCTTGAGAATGTGCCGATA. pLKO.1-puro-shGFP was used as control. To stably overexpress USP15 in ovarian cancer cells, USP15 was amplified by PCR using specific primers (Table S7) and cloned into pLVX-IRES-Gβγ-BERKY3 plasmid (Addgene, Plasmid #158426) using restriction digestion followed by Gibson assembly. Site-directed mutagenesis was performed using pLVX-USP15 template and specific primers (Table S7) to generate USP15 rescue plasmid. Lenti-luciferase-P2A-Neo (Addgene, Plasmid #105621) was used to stably express luciferase in ovarian cancer cells.

### Reagents and antibodies

DMEM (Cat No. 45000-304), RPMI-1640 with L-glutamine (# 45000-396), DMEM/Ham’s F-12 50/50 Mix (# 45000-344) were obtained from VWR. Fetal bovine serum (FBS) was obtained from Cell Culture collectives (# FB-02). Pen-Strep (# 15140163) and crystal violet (# 405831000) was purchased from Thermo Fisher Scientific. Puromycin hydrochloride (# 13884), G418 sulfate (# 13200), ampicillin sodium salt (#14417) were obtained from Cayman Chemicals. Bio-Rad Protein Assay Dye Reagent was obtained from Bio-Rad (# 5000006). WST-1 reagent (# 11644807001) and thymidine (#T1895-1G) was obtained from Sigma-Aldrich. Hoechst 33342 (#H3570) and ProLong glass antifade mountant (#P36982) was purchased from Invitrogen. Around 24-well inserts were purchased from Fisher Scientific (# 07–000–396). Matrigel was obtained from VWR (# 47743-715). FACS tube with a 35 μm strainer Cap was purchased from Stellar Scientific (# FSC-9005) and propidium iodide/RNase solution was obtained from BD Bioscience (# 550825). D-luciferin was purchased from Syd Lab (# MB000102-R70170). The list of antibodies, their source, and the dilutions used in this study are listed in Table S8.

### Cell lines and cell culture conditions

HEK293T cells were cultured in DMEM and supplemented with 10% FBS and 1% Pen-Strep. TYK-Nu, OVCA420, OVCAR3, OVCAR5, and MDAH2774 cells were a kind gift from Dr. K.K. Wong (MD, Anderson Cancer Center, TX, USA). OVCAR8 cells were a kind gift from Dr. Priyanka Verma (Wash U, MO, USA). All human ovarian cancer cells were cultured in RPMI-1640 medium that is supplemented with 10% FBS and 1% Pen-Strep. FT194 cells were a kind gift from Dr. Ronny Drapkin (UPenn, PA, USA) and were cultured in DMEM/Ham’s F-12 50/50 Mix and supplemented with 10% FBS and 1% Pen-Strep. All cells were grown at 37°C in the presence of 5% CO_2_.

### Western blot analysis

Cell lysates were prepared in RIPA buffer supplemented with protease and phosphatase inhibitors. Protein concentrations were determined using Bio-Rad Protein Assay Dye Reagent. Equal amount of total protein was run using an SDS-PAGE gel and then transferred to a polyvinylidene fluoride (PVDF) membrane. The membrane was blocked for 1 h at room temperature in 3% milk, and then incubated with primary antibodies overnight at 4°C on a shaker. The membranes were washed three times with 1*×* TBST and incubated with secondary antibodies for 1 h at room temperature on a shaker. Membranes were washed again with 1*×* TBST three times, developed with enhanced chemiluminescence (ECL) reagent, and imaged with a BioRad ChemiDoc Imager.

### RNA-seq analysis

Flash frozen cell pellets were shipped on dry ice to Azenta Life Sciences. Azenta Life Sciences performed RNA extraction and RNA-seq using their standardized procedure. Three biological replicates were submitted for each sample. Proper quality check was included to ensure data quality. Preliminary data analysis was performed by Azenta Life Sciences as part of the package. Genes with an adjusted *p* value <0.05 and absolute log2 fold change >1 was considered significant. Volcano plot and heatmap were generated using the SRPlot platform.^44^

### Cell viability and proliferation assays

For WST-1 assays 5,000 cells were plated in each well of a 96-well plate in triplicate and allowed to grow at 37°C in CO_2_ incubator. WST-1 assay was performed 48, 72, and 96 h later. Number of viable cells were determine using WST-1 reagent at 24 and 48 h as per manufacturer’s instructions. Colorimetric reading was acquired using BioTek Gen5 plate reader. For crystal violet staining, 5,000–10,000 cells, depending on the cell lines, were seeded in a 6-well plate and allowed to grow for 5–7 days. Cells were then fixed using 4% paraformaldehyde (PFA) and stained with 0.25% crystal violet. Images were taken with a 4*×* objective and quantified using ImageJ software.

### Wound healing assays

Cells were seeded in a 12-well plate and allowed to grow for 24 h at 37°C to allow them to reach 90%–95% confluency. At 24 h, a linear wound was created on the cell monolayer using a 200 μL pipette tip. Detached cells were removed by washing with PBS, and fresh RPMI supplemented with 10% FBS and 1% penicillin-streptomycin was added. The plates were imaged at 4 h intervals using BioTek Cytation 5. Wound closure rate was quantified using ImageJ.

### Transwell migration assays

Approximately, 3–5 × 10^4^ cells were seeded in RPMI containing 10% FBS inside 24-well inserts. RPMI containing 20% FBS was added to the 24-well dish, beneath the permeable insert. The cells were then incubated for 24–48 h at 37°C in 5% CO_2_. Migratory cells were fixed using 4% PFA, stained using 0.25% crystal violet, imaged with a 4*×* objective and quantified using ImageJ.

### Matrigel invasion assays

A 24-well permeable inserts were coated with 40 μL of Matrigel in 1:3 ratio and allowed to set for 1 h at room temperature. Each insert was placed in a well in a 24-well plate containing RPMI media with 20% FBS. 0.5–1 × 10^5^ cells were reconstituted in RPMI media with 10% FBS and added to the inserts and incubated at 37°C in 5% CO_2_ for 24–72 h. Cells that invaded through the Matrigel were fixed with 4% PFA, stained with 0.25% crystal violet, imaged using a 4*×* objective and quantified using ImageJ.

### Immunofluorescence microscopy

Approximately, 1 × 10^5^ cells were seeded onto coverslips and allowed to adhere overnight in the incubator at 37°C with 5% CO_2_. Cells were fixed with 4% PFA for 20 min at room temperature, quenched for 10 min at room temperature and permeabilized using 0.1% Triton X-100 for 10 min. Permeabilized cells were blocked with 4% bovine serum albumin (BSA) in PBS for 1 h at room temperature. Primary antibodies were diluted in 4% BSA/PBS and incubated overnight at 4°C. The primary antibodies were removed the following day and cells were blocked with 4% BSA in PBS at room temperature for 30 min prior to incubation with secondary antibodies (in 4% BSA in PBS) for 45 min at room temperature in the dark. Cells were washed with PBS, stained with Hoechst, mounted on a glass slide, and imaged using the Zeiss Axioscope 5 fluorescence microscope. For confocal microscopy, cells were mounted with ProLong Glass Antifade Mountant and high precision cover glass. Images were collected with a Zeiss LSM 900 confocal with Airyscan 2. Gamma H2A-X images were acquired in confocal imaging mode and anaphase bridge images were acquired in SR (Airyscan) imaging mode. Airyscan images were processed using automatic filter settings. Image analysis was performed using FIJI. Antibodies used are listed in Table S8.

### Cell cycle synchronization

Control and USP15 knockdown cells were grown in RPMI media supplemented with 10% FBS and 1% Pen-Strep to approximately 30% confluency and 2 mM thymidine was added. After 18 h, the thymidine-containing media was removed and cells were washed with PBS. Fresh RPMI media with 10% FBS was added to these cells and the cells were incubated for 9 h at 37°C with 5% CO_2_. Following this incubation, cells underwent a second thymidine treatment for 15 h. Subsequently, the cells were released from the block and washed with PBS. Fresh media was added, and the cells were harvested every 6 h for a total duration of 36 h.

### Flow cytometry

OVCA420 and OVCAR8 cells were grown in RPMI medium supplemented with 10% FBS and 1% Pen-Strep to approximately 40%–60% confluency. Cells were harvested, and 1 × 10^6^ cells were fixed in ice-cold 70% ethanol, stained with a propidium iodide/RNase solution for 30 min in the dark. After incubation, the cells were passed through a 5 mL round bottom polystyrene FACS tube with a 35 μm strainer Cap and analyzed using BD FACS Canto II Flow Cytometry system at the University of Maryland, Baltimore Flow Core. Data were quantified using the FCS Express software. The cell cycle results were analyzed using FCS Express 7 software. Gates were established using the forward scatter and side scatter data. Doublet discrimination was performed using the PI width and PI area to filter out aggregates. Similar gating methods were applied to all groups. The DNA content histogram was generated, and the G1, S, and G2-M fractions were quantified using appropriate models. CoraLite Plus 488-Annexin V and PI Apoptosis Kit (Proteintech, #PF00005) was used to perform Annexin V staining. GraphPad Prism 10.6.1. was used to generate the graphs and calculate statistical significance.

### *In vivo* mouse experiments

*In vivo* studies were conducted using female NSG (NOD.Cg-Prkdc^scid^ Il2rg^tm1Wjl^/SzJ) mouse strains. All *in vivo* experiments were performed under a protocol approved by the Institutional Animal Care and Use Committee at the University of Maryland Baltimore County (protocol number 1947). Cells were stably tagged with luciferase using the Lenti-luciferase-P2A-Neo plasmid. Approximately, 1 × 10^7^ cells were injected intraperitoneally into 6-week-old female NSG mice. Tumor progression was monitored using the IVIS system (PerkinElmer) every 2 weeks. For IVIS imaging, mice were injected intraperitoneally with 150 mg/Kg D-luciferin in PBS. Signal intensity was quantified by Living Image analysis software (PerkinElmer).

### Quantification and statistical analysis

All *in vitro* experiments were performed using at least three biological replicates. Data are presented as mean ± standard deviation. The individual data points for each biological replicate are shown, and the data were plotted using Graphpad Prism 10.6.1. For comparison between two groups, statistical significance was measured using Welch’s two-tailed *t* test. For comparisons between three or more groups, statistical significance was measured using one-way ANOVA test. For experiments involving two independent variables, statistical significance was measured using two-way ANOVA test. The following were considered significant: *p* < 0.05 (∗), *p* < 0.005 (∗∗), *p* < 0.001 (∗∗∗), *p* < 0.0001 (∗∗∗∗). Cell cycle analysis experiments were performed using three biological replicates, and the data were analyzed using the FCS Express software. Statistical significance was determined using Welch’s *t* test, and two-way ANOVA for experiments involving at least two independent variables. Identical parameters were maintained between the respective control and experimental groups to acquire fluorescence images used in this study. The images were analyzed using ImageJ software. RNA-seq was also performed using biological triplicates. Distribution of read counts in libraries was examined before and after normalization. The original read counts were normalized to adjust for various factors, such as variations of sequencing yield between samples. These normalized read counts were used to accurately determine differentially expressed genes. The overall similarity among samples was assessed by the Euclidean distance between samples. This method was used to examine which samples are similar/different from each other and if they fit to the expectation from the experiment design. A comparison of gene expression between the groups of samples was performed using DESeq2. The Wald test was used to generate *p* values and log2 fold changes. Genes with an adjusted *p* value <0.05 and absolute log2 fold change >1 were called as differentially expressed genes. Significantly differentially expressed genes were clustered by their GO and the enrichment of GO terms was tested using Fisher’s exact test (GeneSCF v.1.1-p2). Volcano plots and heatmaps were generated using the SRPlot platform.^44^ For *in vivo* tumor burden experiments in mice, statistical significance was determined using two-way ANOVA followed by Tukey’s multiple comparisons test. Survival studies in mice were analyzed by Kaplan-Meier curves and log rank (Mantel-Cox) tests.

## Data and code availability

All data supporting the findings of this study are available within the article and its supplementary file. RNA-seq data used in the manuscript has been submitted to the Sequence Read Archive (SRA) database and the accession number is PRJNA1508211. There are no restrictions on data availability.

## Acknowledgments

We thank Mr. Pavan Umashankar for help with cloning the USP15 rescue construct. The FT194 cell lines were a gift from Dr. Ronny Drapkin (UPenn). A.P. has been supported by the 10.13039/100000005Department of Defense (HT9425-23-1-0351 and HT9425-23-1-0232), 10.13039/100000002National Institutes of Health (R03CA282712), funds from the 10.13039/100012870Ovarian Cancer Alliance of Greater Cincinnati (PRIV0201 and PRIV0219), and 10.13039/100018233UMGCCC 10.13039/100000048American Cancer Society Institutional Research Grant IRG-18-160-16. A.O. work was supported in part by 10.13039/100000002NIH grant T32 GM158458. We would like to thank Dr. Xiaoxuan Fan and Flow Cytometry Shared Service of the University of Maryland Marlene and Stewart Greenebaum Comprehensive Cancer Center for help with cell cycle analysis. This publication was supported by funds through the Maryland Department of Health's Cigarette restitution fund program and the National Cancer Institute-Cancer Center Support grant (CCSG) P30CA134274.

## Author contributions

A.O., experimental design, reagent development, investigation, data analysis and interpretation, writing – original draft, and writing – review and editing; F.A., investigation, data analysis and interpretation, writing – original draft, and writing – review and editing; O.N.A., reagent development, investigation, and writing – review and editing; A.P., conceptualization, experimental design, reagent development, investigation, data analysis and interpretation, writing–original draft, writing–review and editing, funding acquisition, and supervision.

## Declaration of interests

The authors declare no competing interests.

## Supplemental information

Document S1. Figures S1–S5 and Tables S7 and S8

Table S1. List of differentially altered genes between control and USP15 knockdown TYK-Nu cells

Table S2. List of differentially altered genes between control and USP15 knockdown OVCA420 cells

Table S3. List of differentially altered genes between control and USP15 knockdown OVCAR8 cells

Table S4. Genes that are differentially altered in both TYK-Nu and OVCA420 cells upon USP15 knockdown

Table S5. Genes that are differentially altered in both TYK-Nu and OVCAR8 cells upon USP15 knockdown

Table S6. Genes that are differentially altered in both OVCA420 and OVCAR8 cells upon USP15 knockdown

Document S2. Article plus supplemental information
