# Supplementary material for: USP15 regulates mitotic fidelity, metastatic potential, and chemotherapeutic response in ovarian cancer cells
Source: Mol Ther Oncol. 2026 Aug 20;34(3):201329. doi: 10.1016/j.omton.2026.201329 (PMC13571140; doi:10.1016/j.omton.2026.201329)
Supplement: Supplementary file 1 [file mmc1.pdf]

OMTON, Volume *34*

Supplemental information

USP15 regulates mitotic fidelity, metastatic potential, and chemotherapeutic response in ovarian cancer cells

Ayokunnumi Ogunsanya, Fatimah Alfaran, Omuya Noel Amadu, and Achuth Padmanabhan

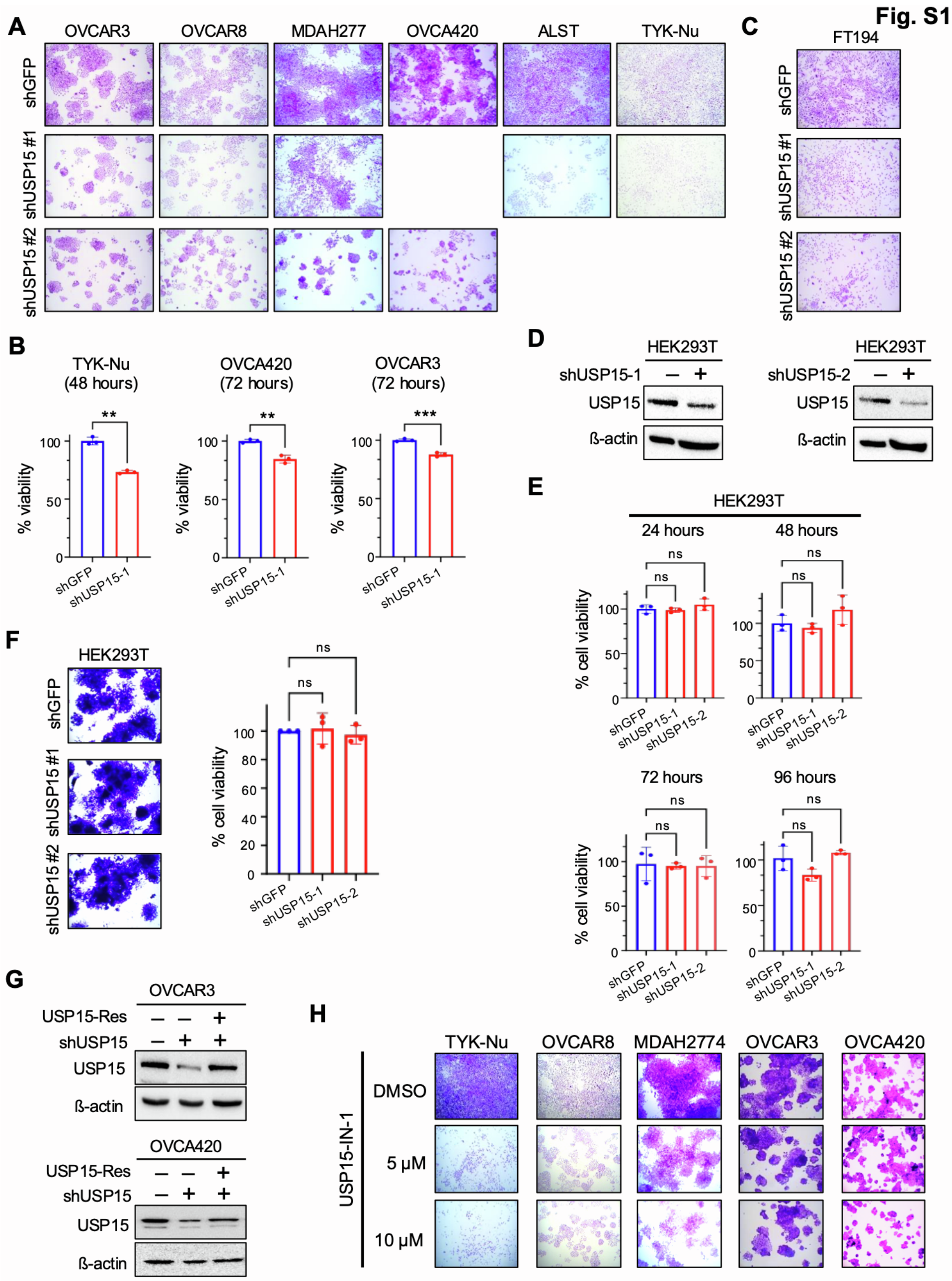

Figure S1. USP15 depletion reduces ovarian cancer cell viability.

(A) Crystal violet staining showing USP15 depletion in ovarian cancer cells causes significant reduction in cell viability and proliferation. **(B)** WST-1 assay show that USP15 depletion causes reduction in cell viability in TYK-Nu, OVCA420, and OVCAR3 cells. **(C)** Crystal violet staining showing USP15 depletion in the immortalized fallopian tube

secretory epithelial cell line FT194 causes reduction in cell viability and proliferation. **(D)** Western blot confirming USP15 knockdown in HEK293T cells. **(E)** WST-1 assay show that USP15 depletion does not impact cell viability in HEK293T cells. **(F)** Crystal violet staining showing long-term (8 days) USP15 depletion does not impact cell viability and proliferation in HEK293T cells. **(G)** Western blot confirming rescue of USP15 expression in USP15 knockdown OVCAR3 and OVCA420 cell lines. **(H)** Crystal violet staining showing USP15 inhibition causes decrease in viability and proliferation ovarian cancer cells.

A **Fig. S2**

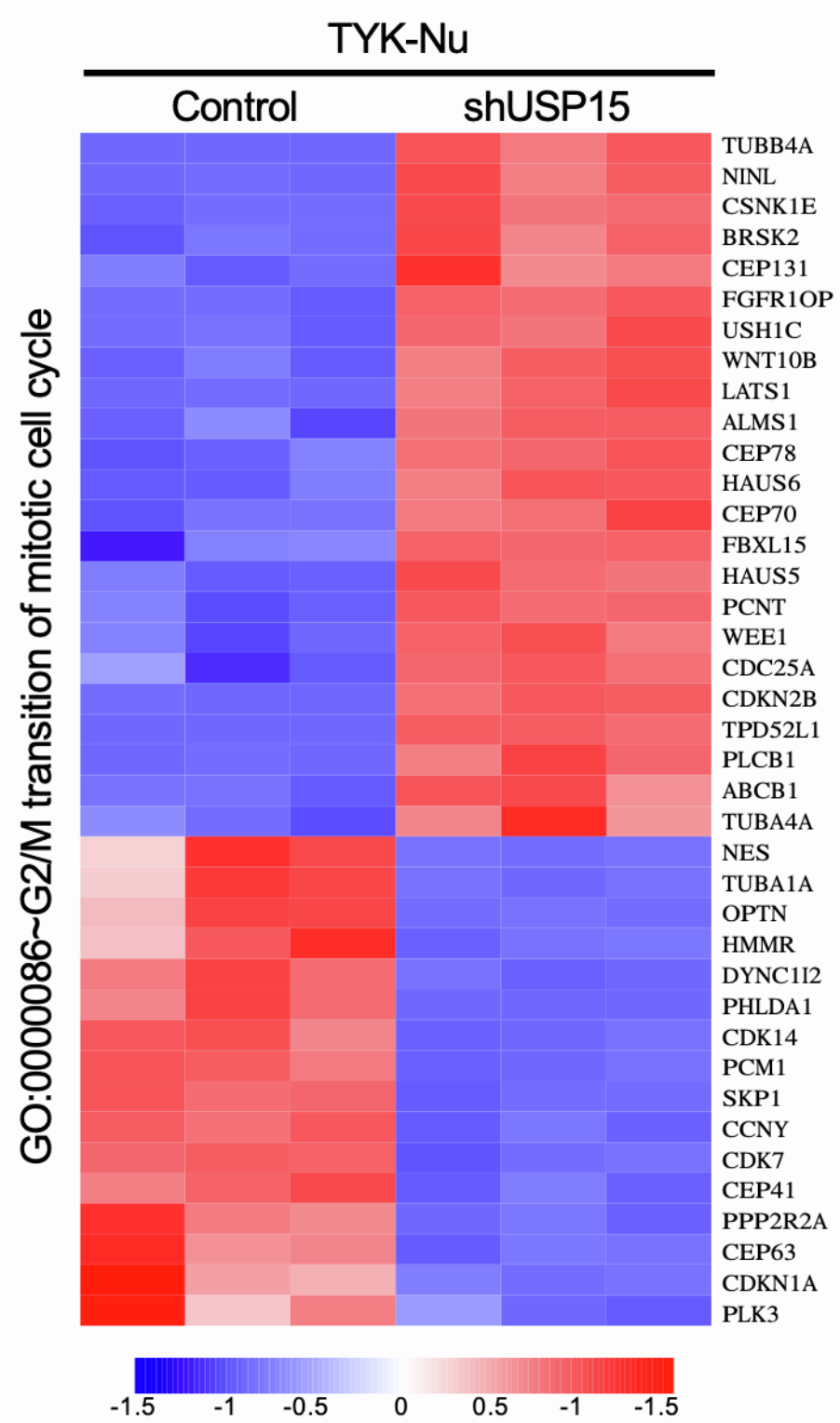

Figure S2. USP15 regulates transition of ovarian cancer cells through the G2-M checkpoint.

(A) Heatmap showing differences in the expression of genes involved in G2-M transition in TYK-Nu cells upon USP15 knockdown.

Fig. S3

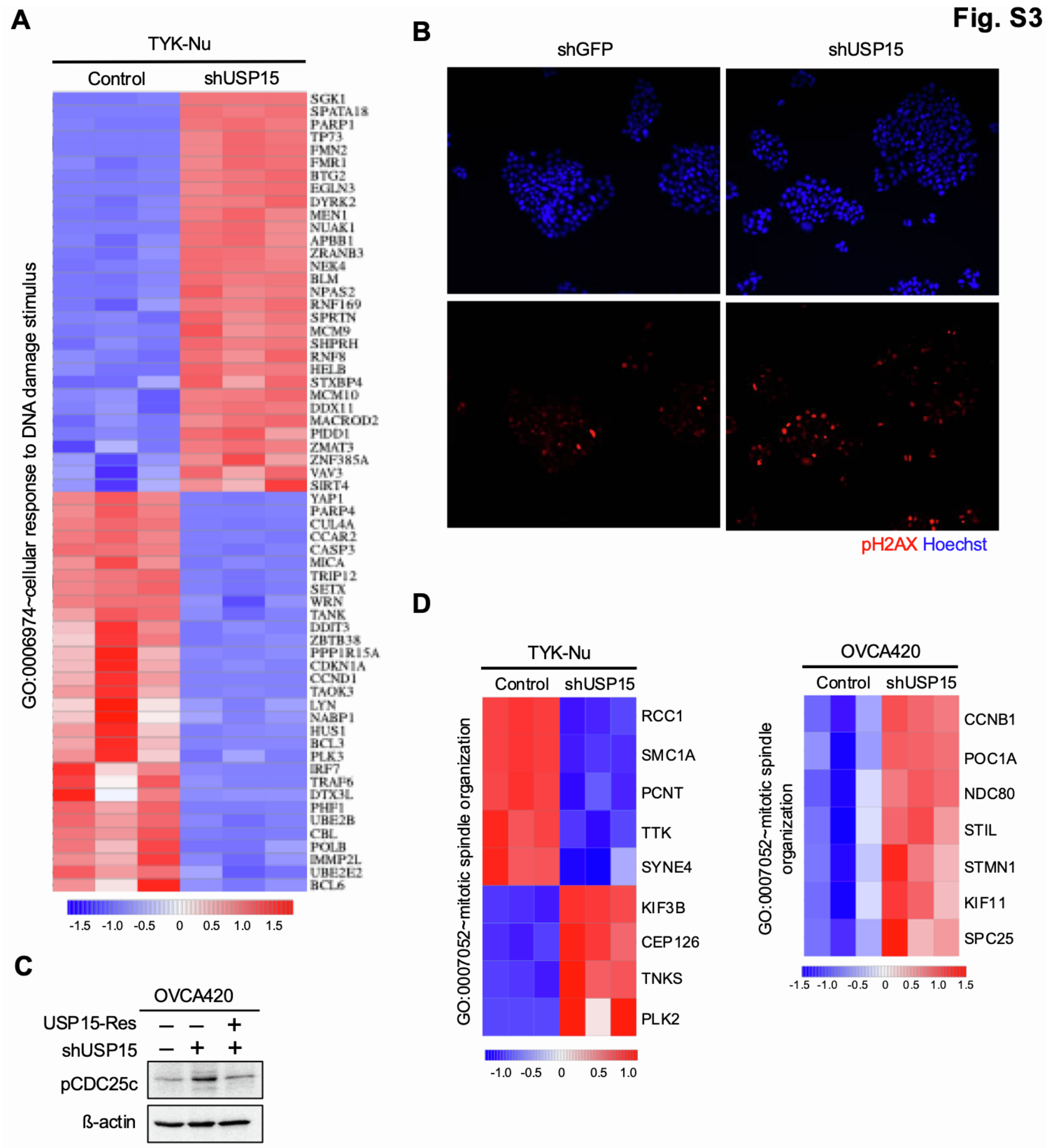

Figure S3. USP15 depletion induces chromosome segregation defects and DNA damage.

(A) Heatmap showing differences in the expression of genes that are involved in cellular response to DNA damage stimulus (GO term) in TYK-Nu cells upon USP15 knockdown. **(B)** Representative immunofluorescence images showing elevated γH2AX staining (red) in USP15 depleted OVCA420 cells compared to control cells. Nucleus (Hoechst stain) is shown in blue. **(C)** Western blot showing the effect of re-expressing USP15 in USP15 knockdown OVCA420 cell on phospho-Cdc25c. **(D)** Heatmap from RNAseq data showing

genes involved in mitotic spindle organization that are differentially expressed up on USP15 depletion in TYK-Nu (left) and OVCA420 (right) cells.

Fig. S4

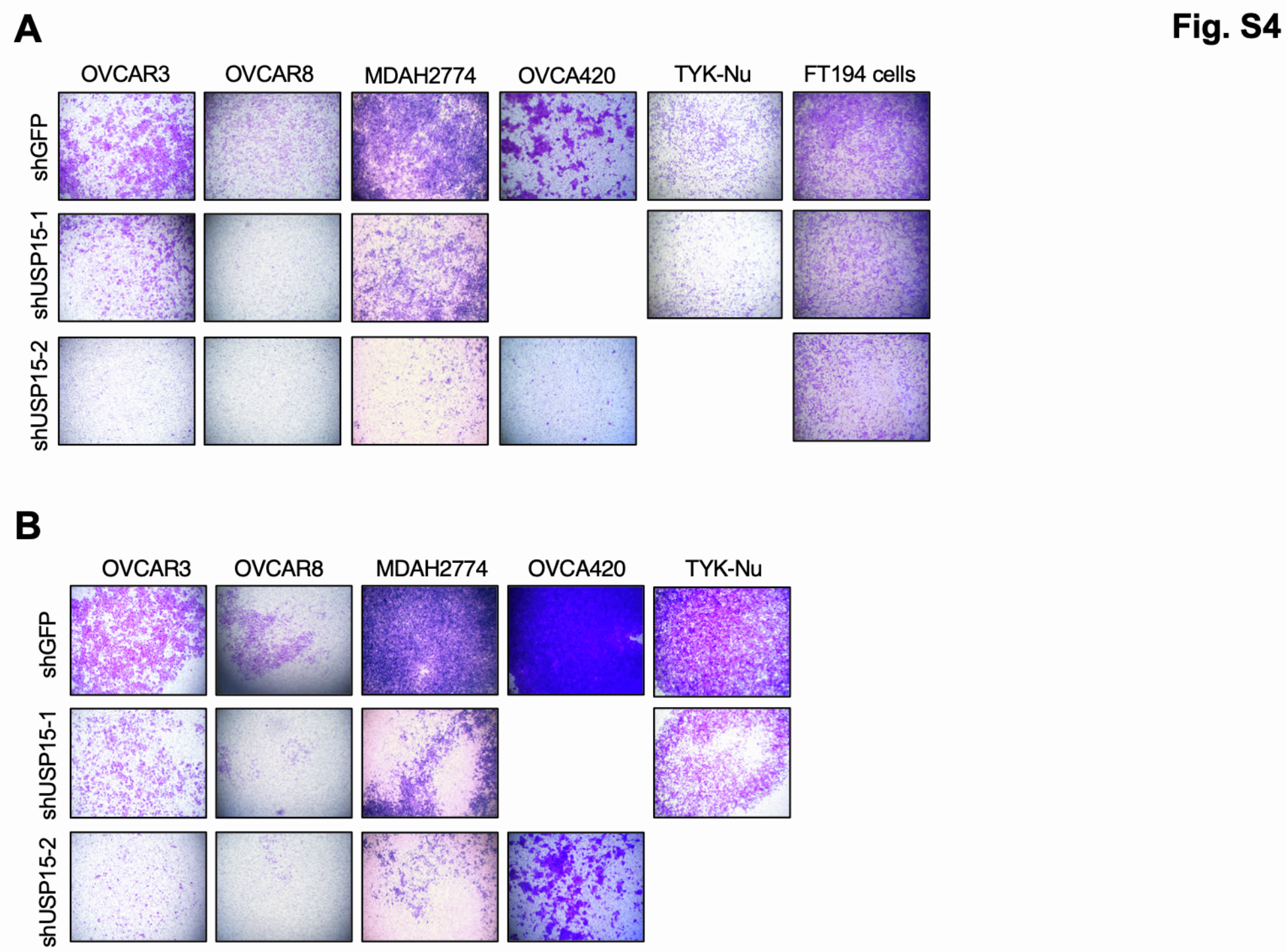

Figure S4. USP15 impacts the metastatic potential of ovarian cancer cells.

(A) Representative crystal violet staining show that USP15 depletion in ovarian cancer cell lines as well as the fallopian tube cell line FT194 causes significant reduction in their ability to migrate in transwell migration assay. **(B)** Representative crystal violet staining show that USP15 depletion in ovarian cancer cells causes significant reduction in their ability to invade through matrigel.

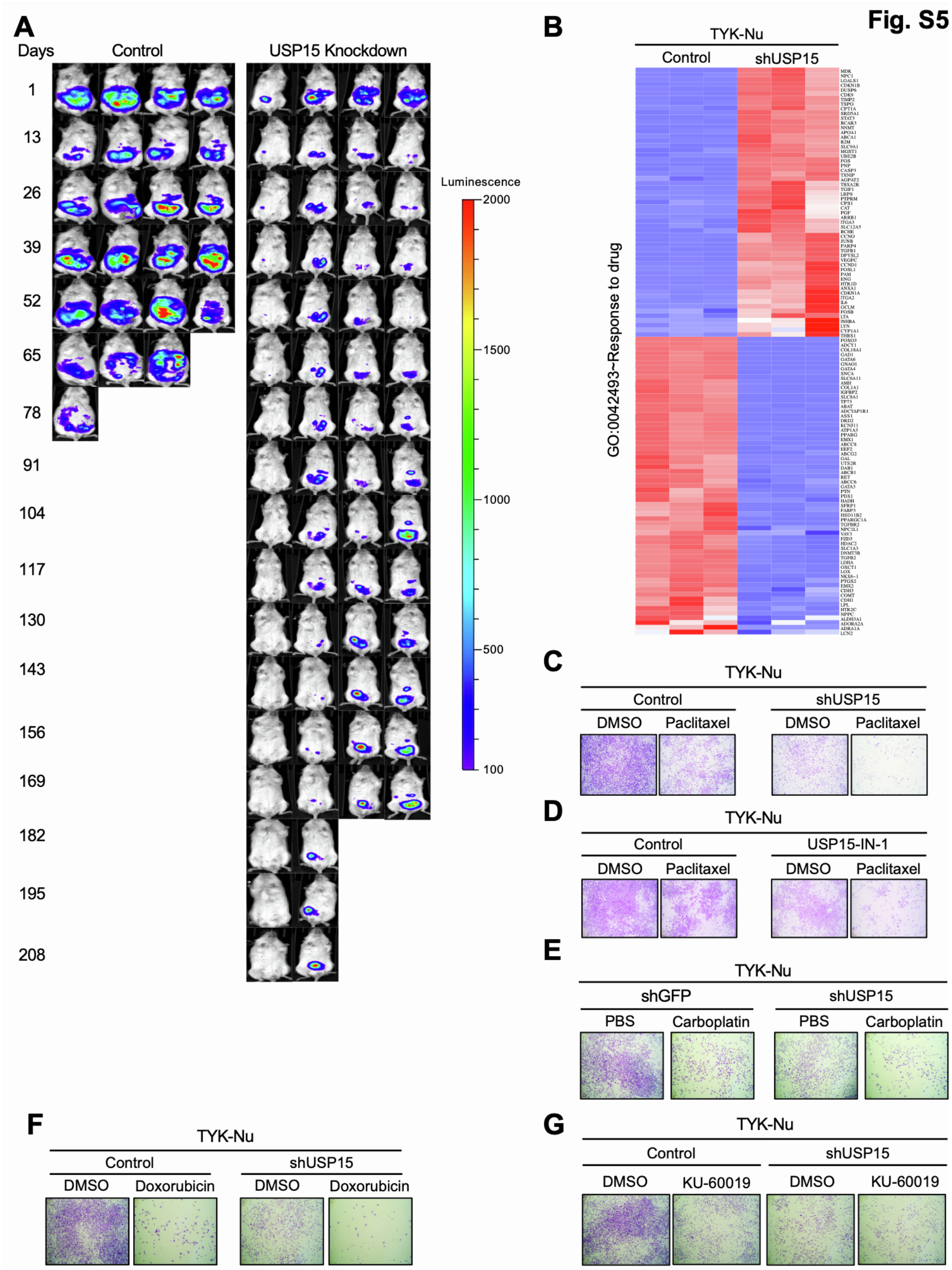

Figure S5. USP15 depletion sensitizes ovarian cancer cells to chemotherapeutics.

(A) Representative in vivo luciferase images form NSG mice intraperitoneally injected with either control or USP15 knockdown TYK-Nu cells. **(B)** Heatmap from RNAseq data showing genes involved in Response to drug (GO term) that are differentially expressed up on USP15 depletion in TYK-Nu cells. **(C)** Representative crystal violet staining

showing USP15 depletion sensitizes TYK-Nu cells to paclitaxel (0.5 nM; 6 days). **(D)** Representative crystal violet staining showing USP15 inhibitor, USP15-IN-1 (2.5 μM) sensitizes TYK-Nu cells to paclitaxel (0.5 nM; 6 days). **(E)** Representative crystal violet staining showing USP15 depletion sensitizes TYK-Nu cells to carboplatin (10 μM; 6 days). **(F)** Representative crystal violet staining showing USP15 depletion sensitizes TYK-Nu cells to doxorubicin (100 nM; 6 days). **(G)** Representative crystal violet staining showing USP15 depletion sensitizes TYK-Nu cells to KU-60019 (5 μM; 6 days).

Supplemental Tables

Table S1. List of differentially altered genes between Control and USP15 knockdown TYK-Nu cells. (see attached MS Excel file)

Table S2. List of differentially altered genes between Control and USP15 knockdown OVCA420 cells. (see attached MS Excel file)

Table S3. List of differentially altered genes between Control and USP15 knockdown OVCAR8 cells. (see attached MS Excel file)

Table S4. Genes that are differentially altered in both TYK-Nu and OVCA420 cells upon USP15 knockdown. (see attached MS Excel file)

Table S5. Genes that are differentially altered in both TYK-Nu and OVCAR8 cells upon USP15 knockdown. (see attached MS Excel file)

Table S6. Genes that are differentially altered in both OVCA420 and OVCAR8 cells upon USP15 knockdown. (see attached MS Excel file)

Table S7. List of primers used in this study.

Target	Source	Catalog Number
USP15 (human) Forward Primer	Thermo Fisher	TATTTCCGGTGAATTCCATGTACCCATACGATGTTCCAGATTACGCTGCGGAAGGCGGAGCGGCGGA
USP15 (human) Reverse Primer	Thermo Fisher	GGGAGAGGGGCGGGATCCGCTTAGTTAGTGTGCATACAGT
USP15 Rescue Construct Forward Primer	Thermo Fisher	AGGAGATTATAAAGTTTGTCTTCAGTATTGTTTCGTGGTACAGCC

USP15 Rescue Construct<br><br>Reverse Primer	Thermo Fisher	CCTGTTAAGGATGTGTCGGTATGTCAAAATATCT ACTG

Table S8. List of antibodies used in this study.

Target	Source	Catalog Number	Dilution
ATM	Protein Tech	27156-1-AP	1:500(WB)
ß-actin	Sigma-Aldrich	A2228	1:10000 (WB)
Bub1	ProteinTech	13330-1-AP	1:500 (WB)
CHEK2	ProteinTech	113954-1-AP	1:500(WB)
Cyclin B1	ProteinTech	55004-1-AP	1:1000(WB) 1:200(IF)
E-cadherin	ProteinTech	60335-1-Ig	1:2000(WB)
FITC	Protein Tech	SA00003-2	1:700 (IF)
KIF15	ProteinTech	82903-1-RR	1:2000(WB)
KI67	ProteinTech	27309-1-AP	1:400(WB)
MMP-2	ProteinTech	10373-2-AP	1:500(WB)
MMP-9	ProteinTech	10375-2-AP	1:500(WB)
PARP1	ProteinTech	13371-1-AP	1:1000(WB)
PLK1	ProteinTech	10305-1-AP	1:1000 (WB)
p-Cdc25	Cell Signaling	4901S	1:1000(WB)
Phospho-Histone H2A.X-Ser139	Cell Signaling	9718	1:1000 (IF)
Phospho-Histone H3-Ser10	Cell Signaling	9701S	1:1000 (IF)
Rhodamine (TRITC)-conjugated Goat Anti-Rabbit IgG(H+L)	Protein Tech	SA00007-2	1:400 (IF)

TOP2A	ProteinTech	20233-1-AP	1:2000(WB)
USP15	Cell Signaling	66310	1:1000(WB)
